# Supplementary material for: RNA transcripts serve as a template for double-strand break repair in human cells
Source: Nat Commun. 2025 May 10;16:4349. doi: 10.1038/s41467-025-59510-x (PMC12065846; doi:10.1038/s41467-025-59510-x)
Supplement: Supplementary file 1 — Supplementary Information [file 41467_2025_59510_MOESM1_ESM.pdf]

RNA TRANSCRIPTS SERVE AS A TEMPLATE FOR DOUBLE-STRAND BREAK REPAIR IN HUMAN CELLS

SUPPLEMENTARY INFORMATION

Supplementary Figure 1

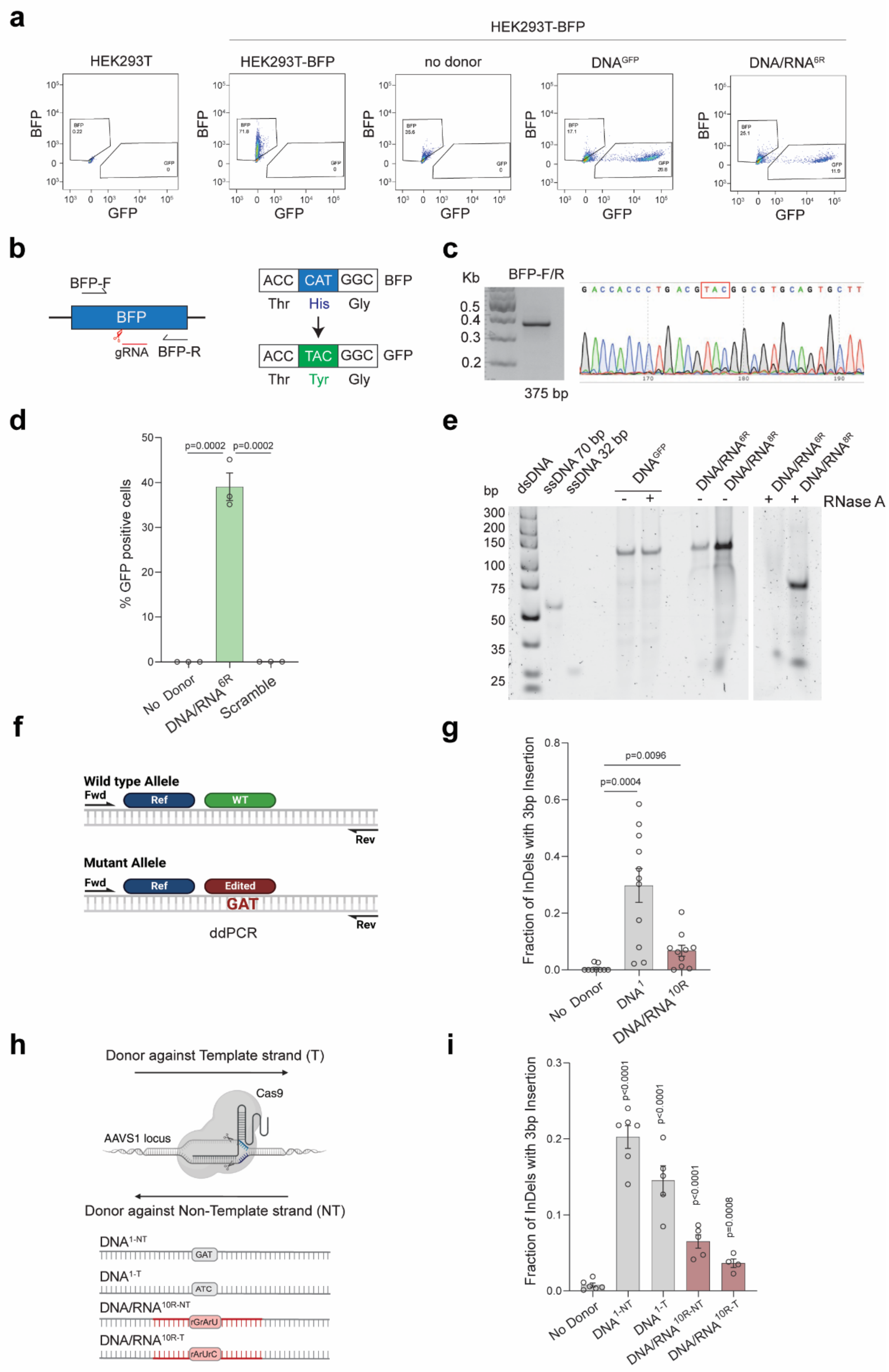

Supplementary Figure 1. Human cells use RNA-containing oligos to repair DSBs.

a, Examples of FACS gating for BFP-to-GFP assay. **b-c**, Schematic of the BFP-to-GFP assay showing the CAT to TAC mutation that induces the BFP to GFP switch. Arrows indicate the primers used to amplify the region around the swap codon, and the resulting PCR band of 375 bp is shown on the left. Sanger sequencing profile confirms the mutated population. **d**, BFP-to-GFP assay, in clonally derived *TP53BP1*$^{-/-}$ cells described in Supplementary Fig. 3 e-f, using a scramble donor containing a stop sequence (DNA/RNA$^{Stop\text{-}6R}$) in the RNA portion instead of the amino acid switch codon (n=3 biological replicates). **e**, Native PAGE gel showing the purity of donors used in Fig.1b. Double-stranded (dsDNA) and single-stranded (ssDNA) were loaded as references. The DNA/RNA$^{6R/8R}$ donors were cleaved with RNaseA, and the DNA^{GFP} donor was used as a negative control. **f**, Schematic of the probes and primers used for the ddPCR to detect the wild type (WT) or mutant allele (Edited) in the AAVS1-seq assay. **g**, Quantification of the relative fraction of droplets with 3bp insertion measured with ddPCR after AAVS1-seq (n=10 biological replicates). **h**, Schematic of AAVS1-seq assay using as donors either the template strand (DNA$^{1\text{-}T}$ and DNA/RNA$^{10R\text{-}T}$) or the non-template strand (DNA$^{1\text{-}NT}$ and DNA/RNA$^{10R\text{-}NT}$). **i**, AAVS1-seq measures the percentage of repair products containing the mutational signature after the Cas9 DSB is repaired in the presence of no donor or the donors from panel G (n=4-6 biological replicates). For d, g, and i, statistical significance was assessed using unpaired two-tailed *t*-tests. Error bars represent the standard error of the mean (± SEM). Schematics were created in BioRender. Jalan, M. (2025) https://BioRender.com/9tmc1xb. Source data are provided as a Source Data file.

Supplementary Figure 2

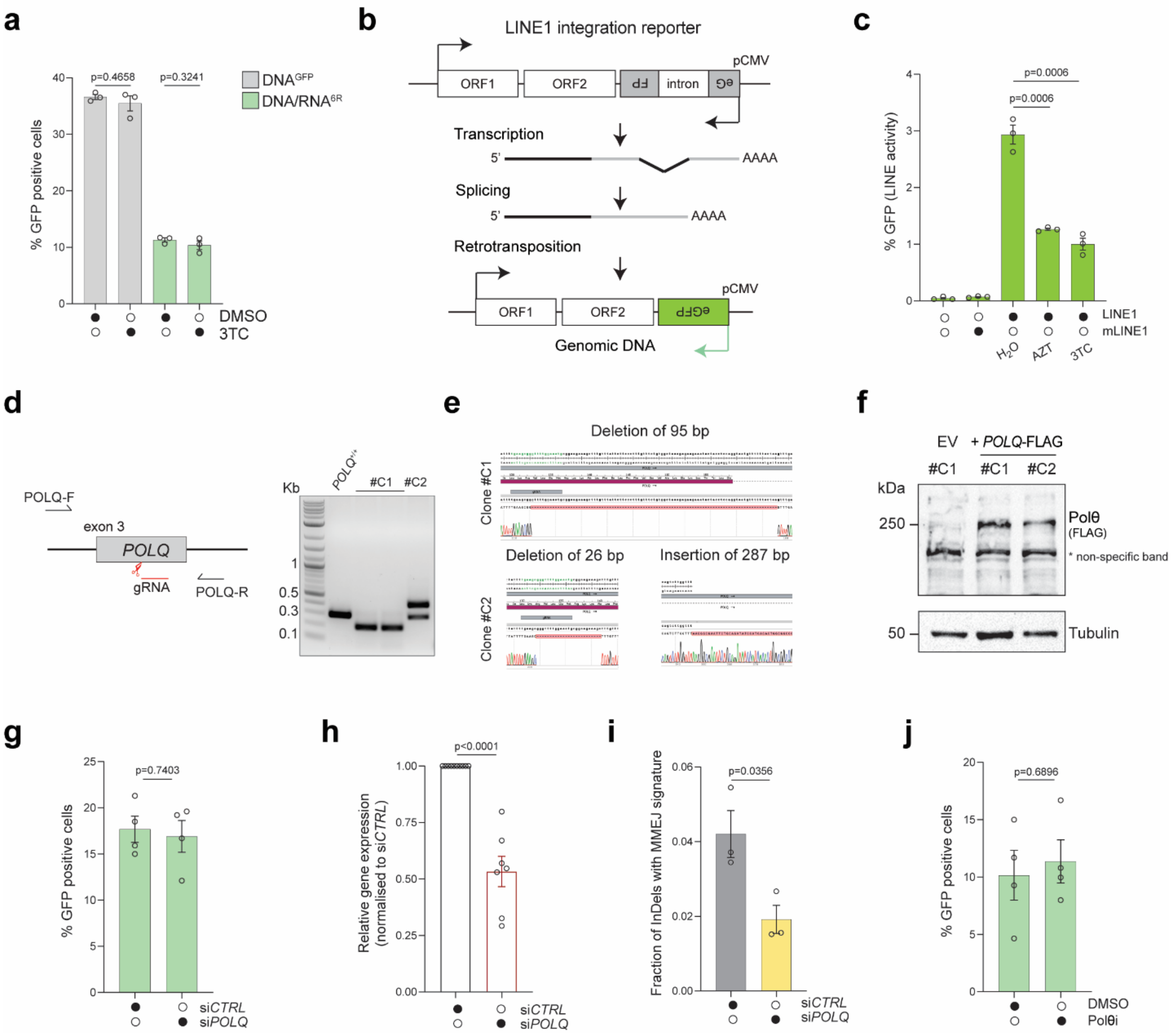

Supplementary Figure 2. RT-DSBR is independent of LINE-1 and Polθ activity.

a, BFP-to-GFP assay with DNA^{GFP} and DNA/RNA^{6R} donors in the presence of 10 µM of the HIV reverse transcriptase inhibitor lamivudine (3TC) or DMSO as a control (n=3 biological replicates). **b**, A plasmid encodes a full-length, retrotransposition-competent LINE-1 element driven by its natural promoter. An EGFP retrotransposition reporter cassette is inserted in the LINE-1 3′ UTR, with the EGFP gene in reverse orientation to the LINE-1 sequence. The sequence is interrupted by an intron in the same orientation as LINE-1. Transcription from the LINE-1 promoter produces an mRNA that does not express EGFP due to the reverse orientation, but if retrotransposition occurs and LINE-1 integrates into the genome, a correctly oriented EGFP mRNA is transcribed from the CMV promoter, resulting in EGFP expression. A control plasmid (mLINE-1), containing a point mutation in ORF1 to disable retrotransposition, is used as a negative control. **c**, HEK293T-BFP cells were incubated with 10 µM of AZT or 3TC 24 hours before transfection with the LINE-1 retrotransposition reporter plasmid and kept in cells throughout the experiment. Transfectants

were selected with puromycin and analyzed by flow cytometry five days post-transfection (n=3 biological replicates). **d**, Schematic of *POLQ* knock-out strategy. DNA gel for genotyping PCR showing the results of CRISPR-Cas9 targeting in two clones. *POLQ*$^{+/+}$ untargeted cells were used as a negative control (n=1 biological replicate). **e**, Clones #C1 and #C2 showed insertion and deletion by Sanger sequencing. **f**, Western blot analysis of POLQ$^{-/-}$ clones and HEK293T-BFP cells transfected with full-length POLQ-FLAG plasmid. Tubulin was used as a loading control (n=1 biological replicate). **g**, BFP-to-GFP assay with DNA/RNA^{6R} donor after knockdown of *POLQ* with siRNA (n=4 biological replicates). **h**, qPCR analysis of *POLQ* mRNA expression after siRNA knockdown of the samples analyzed in main Fig. 2d and Supplementary Fig. 2g (n=7 biological replicates). Relative gene expression was normalized using *ACTB* as a housekeeping gene. **i**, Relative fraction of indels with microhomology-mediated end-joining signature (12 bp deletion with five bp microhomology) after knockdown of *POLQ* through siRNA in the AAVS1-seq assay performed in main Fig. 2d (n=3 biological replicates). **j**, BFP-to-GFP assay with DNA/RNA^{6R} donor after treatment with the Polθ inhibitor RP6685 (n=3 biological replicates). For a, c, g-h statistical significance was assessed using unpaired two-tailed *t*-tests. Error bars represent the standard error of the mean (± SEM). Source data are provided as a Source Data file.

Supplementary Figure 3

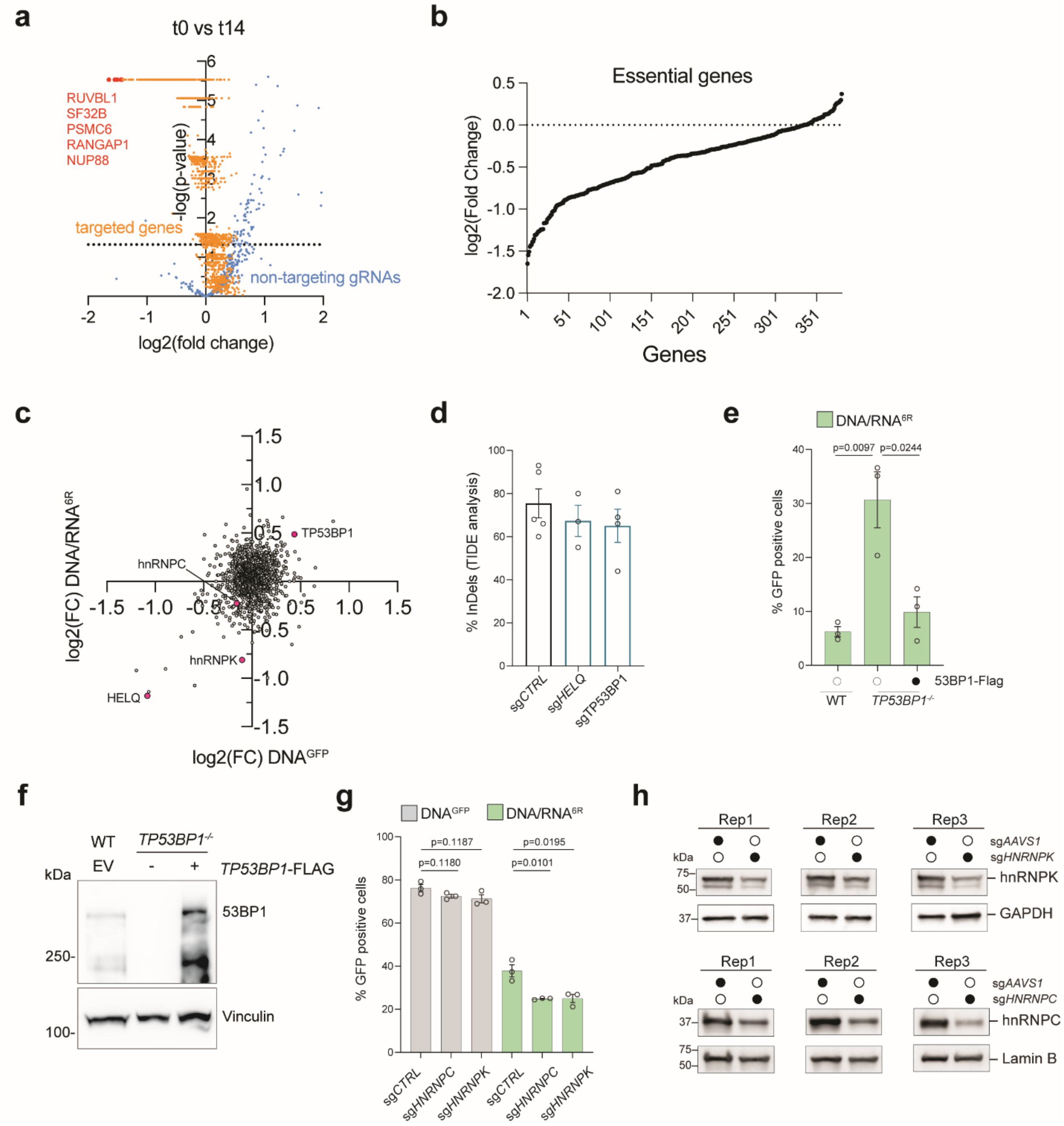

Supplementary Figure 3. A CRISPR/Cas9 screen identifies factors involved in RT-DSBR.

a, Volcano plot depicting genes from the DNA repair library enriched or depleted in the CRISPR/Cas9 screen and gRNA controls. Representative essential genes are highlighted in red, negative control untargeted gRNAs are shown in blue, and targeted genes are in orange. Data were generated from two independent BFP-to-GFP assays performed on the same infected cells: n=2 technical duplicates. **b**, Plot representing log2 fold change in the CRISPR/Cas9 screen comparing t=0 versus t=14 days for 360 genes in the DDR library considered as "common essential"[1]. **c**, Comparison of the log2 fold change for each gene in the CRISPR/Cas9 screen with

the DNA/RNA^{6R} or the DNA^{GFP} donors. **d**, Editing efficiency calculated with TIDE analysis[2] for sgRNAs in Fig. 3c (n=3-5 biological replicates). **e**, BFP-to-GFP assay in a *TP53BP1*$^{-/-}$ clone rescued by 53BP1 overexpression (53BP1-FLAG) (n=3 biological replicates). **f**, Western blot of cells deleted for *TP53BP1* (second lane) and complemented with 53BP1-FLAG (third lane) (n=4 biological replicate). **g**, BFP-to-GFP assay in cells depleted for hnRNPK or hnRNPC in *TP53BP1*$^{-/-}$ cells via the DNA^{GFP} and the DNA/RNA^{6R} donors (n=3 biological replicates). **h**, Western blot of cells depleted for *HNRNPK* and *HNRNPC* with sgRNAs (n=3 biological replicates). For d, e and g statistical significance was assessed using unpaired two-tailed *t*-tests. Error bars represent the standard error of the mean (± SEM). Source data are provided as a Source Data file.

Supplementary Figure 4

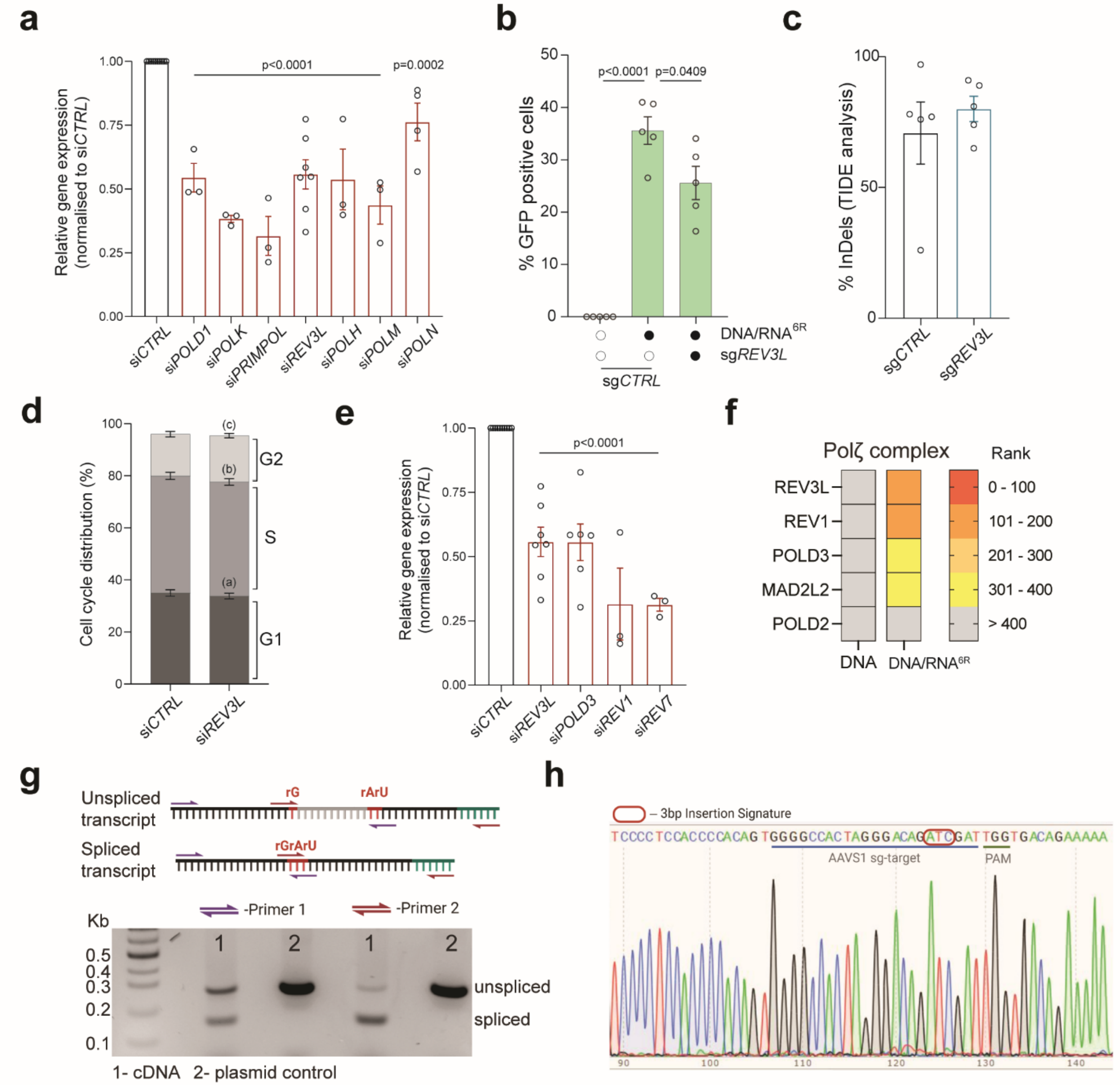

Supplementary Figure 4. The TLS DNA polymerase zeta (ζ) is a reverse-transcriptase *in vivo.*

a, Gene expression analysis by qPCR for siRNA knockdown of the major DNA polymerases in Fig. 4a. *ACTB* was used as a housekeeping gene (n=3-7 biological replicates). **B**, Effect of *REV3L* knockout via sgRNA on the BFP-to-GFP assay using the DNA/RNA^{6R} donor in the *TP53BP1*$^{-/-}$ clone (n=5 biological replicates), compared to a knockout control (sgAAVS1). **c**, Editing efficiency of sgRNA against *REV3L* in Supplementary Fig. 4b calculated with TIDE (n=5 biological replicates). **d**, Comparison of the cell cycle distribution of cells treated with and without si*REV3L* (n=5 biological replicates) [(a) p = 0.4936 (b) p = 0.5643 (c) p = 0.2393] **e**, Gene expression analysis by qPCR of siRNA knockdown of the subunits of Polζ analyzed in Fig. 4d. *ACTB* was used as a housekeeping gene (n=3-7 biological replicates). Error bars represent the standard

error of the mean. **f**, Heatmap of Polζ subunits showing their rank in the screen. **g**, Schematic of the transcribed RNA with the location of the primer pairs used to validate splicing of the transcript donor RNA containing the 3bp mutation signature (See Methods). Gel image showing the spliced and unspliced RNA species amplified from (1) cDNA from cells expressing the plasmid that codes for the donor RNA. (2) Plasmid control with band corresponding to the unspliced RNA product (n=2 biological replicates). **h**, Sanger sequencing profile of the spliced species containing the mutational signature is shown. For a, d and e, statistical significance was assessed using unpaired two-tailed *t*-tests. For b, statistical significance was assessed using 2-way ANOVA. Error bars represent the standard error of the mean (± SEM). Schematics were created in BioRender. Jalan, M. (2025) https://BioRender.com/9tmc1xb. Source data are provided as a Source Data file.

Supplementary Figure 5

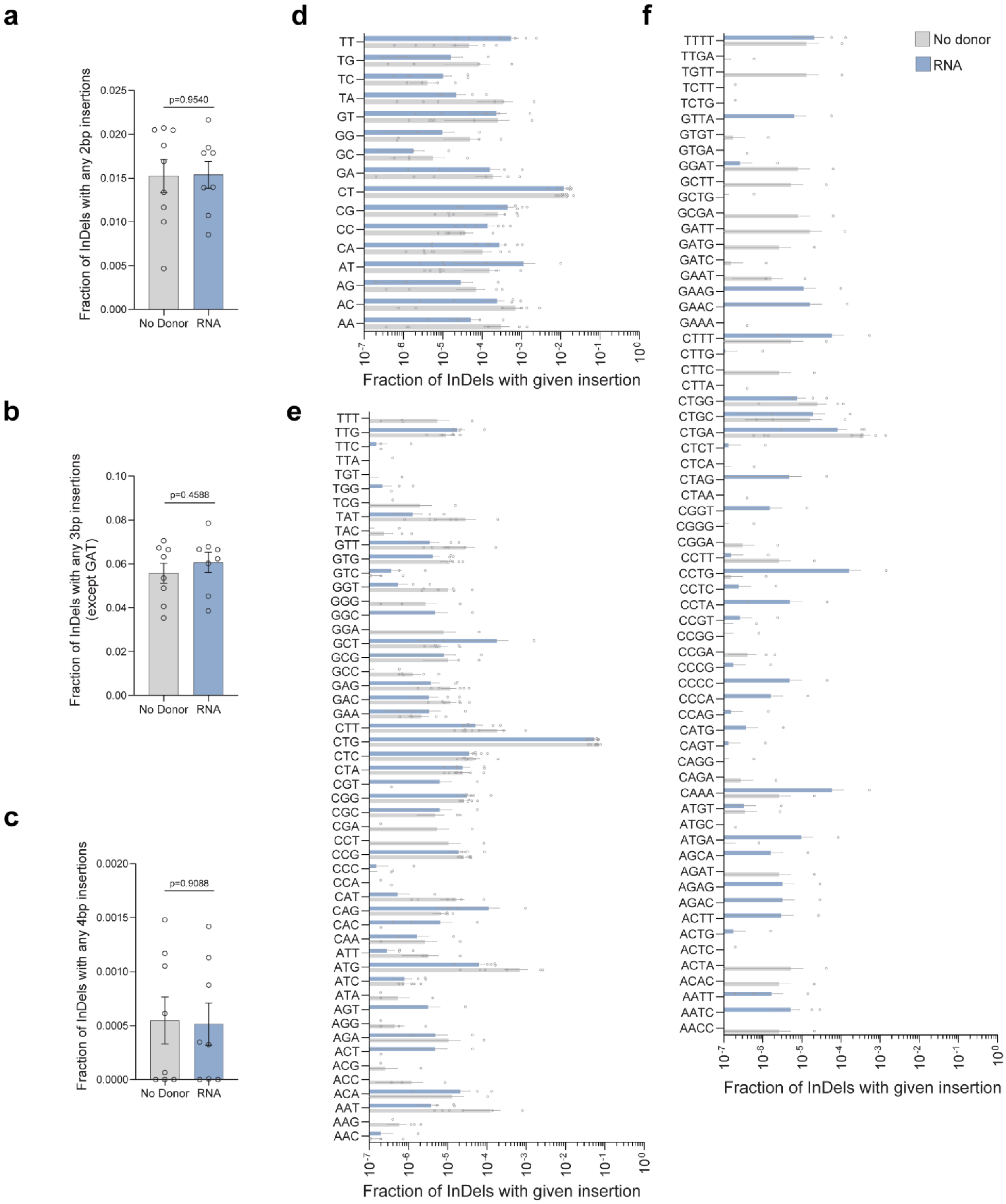

Supplementary Figure 5. Repair via transcript RNA as a donor for RT-DSBR is specific to the template.

Fraction of repair products containing random insertions: (**a**) 2 bp, (**b**) 3 bp (excluding GAT), and (**c**) 4 bp insertions following Cas9-induced breaks repaired with either no donor (n=9 biological replicates) or transcript RNA containing the GAT mutational signature (n=8 biological replicates), as measured by RT-DSBR. Statistical significance was assessed using an unpaired two-tailed *t*-tests. Error bars represent the standard error of the mean (± SEM). **d**, **e**, and **f** show the frequency of individual insertional signatures contributing to the overall values presented in **a**, **b**, and **c,** respectively. Error bars represent the standard error of the mean (± SEM). Source data are provided as a Source Data file.

Supplementary Figure 6

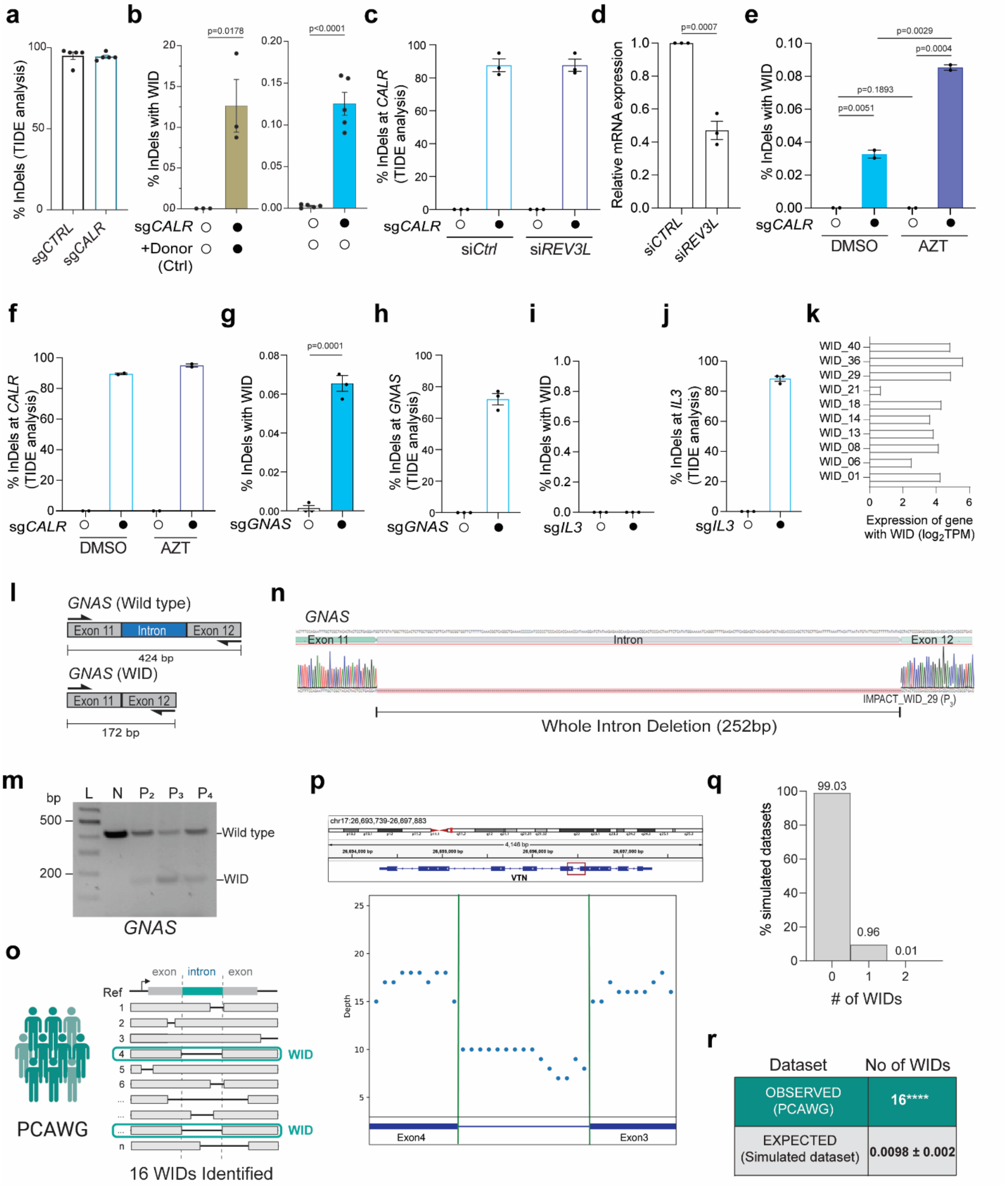

Dataset	No of WIDs
OBSERVED (PCAWG)	16****
EXPECTED (Simulated dataset)	0.0098 ± 0.002

Supplementary Figure 6. Repair via spliced RNA as a donor for RT-DSBR leads to WIDs.
a, Editing efficiency for sgRNA targeting *CALR* intron 2 calculated with TIDE analysis[2] (n=3 biological replicates). **b**, Quantification of reads containing a precise deletion of CALR intron 2 as a fraction of total reads after CRISPR-Cas9-induced break (n=3-5 biological replicates). Left graph: WIDs were quantified in cells transfected with a donor lacking the intron, compared to control cells without DSB induction. Right graph: A CRISPR-Cas9-mediated DSB was introduced without any exogenous donor template provided. WIDs driven by endogenous spliced mRNA were measured as a fraction of repair events. **c**, Editing efficiency for sgRNA targeting *CALR* intron 2 as depicted in Fig. 5b (n=3 biological replicates). **d**, qPCR to quantify the relative levels of *REV3L* transcripts in cells treated with control siRNA and siREV3L (n=3 biological replicates). **e**, Quantification of reads containing precise WIDs (as a fraction of total repair events) at *CALR* intron 2 in control cells (DMSO treated) and ones treated with LINE1 inhibitor AZT (n=2 biological replicates) with and without a CRISPR/Cas9-mediated DSB. **f,** TIDE analysis confirming efficient CRIPSR-Cas9 cleavage at *CALR* intron 2 (n=2 biological replicates). **g**, Quantifying WIDs in cells treated with sgRNA targeting *GNAS* intron 11 (n=3 biological replicates)**. h,** TIDE analysis confirming efficient CRIPSR-Cas9 cleavage at *GNAS* intron 11 (n=3 biological replicates). For a-j, statistical significance was assessed using unpaired two-tailed *t*-tests. Error bars represent the standard error of the mean (± SEM). **i**, Quantifying WIDs in cells treated with sgRNA targeting the non-transcribed control gene, *IL3* intron 4 (n=3 biological replicates). **j**, TIDE analysis confirming efficient CRIPSR-Cas9 cleavage at *IL3* intron 4 (n=3 biological replicates). **k**, Relative expression levels for the genes containing the WIDs in the tumor samples as determined by RNA-Seq analysis. **l**, Schematic of the exons spanning the WID in *GNAS* with the flanking primers used to confirm the sequence. A 172bp deletion was observed for the *GNAS* gene that maps precisely to the intron flanked by Exon 11-12. **m**, Agarose gel depicting the full-length band corresponding to the locus spanning Exon 11-12 in normal MCF-12A cells (N) and the shorted locus with the intron loss in the tumor sample in GNAS. P_2-P_4 represents three different patients from the MSK-IMPACT cohort (n=1 biological replicate). **n**, Sanger sequencing of the PCR products to confirm the presence of the WID in *GNAS*. **o**, Schematic of the pipeline to analyze the deletion profiles of PCAWG tumors. **p**, Example of a WID found in the *VTN* gene of a patient sample from the PCAWG cohort. **q**, 10,000 PCAWG-like cohorts were simulated, and the occurrence of WIDs was calculated. Graph representing the number of WID observed in the PCAWG-like simulated datasets. **r,** The number of expected WID was calculated after randomization of the deletion locations across the whole genome. Using two tailed Fisher's exact test, empirical *p*-values were calculated by comparing the observed versus the 10,000 random values. (**** $p < 0.0001$). Schematics were created in BioRender. Jalan, M. (2025) https://BioRender.com/9tmc1xb. Source data are provided as a Source Data file.

REFERENCES

1. Hart T*, et al.* High-Resolution CRISPR Screens Reveal Fitness Genes and Genotype-Specific Cancer Liabilities. *Cell* **163**, 1515-1526 (2015).

2. Brinkman EK, Chen T, Amendola M, van Steensel B. Easy quantitative assessment of genome editing by sequence trace decomposition. *Nucleic Acids Res* **42**, e168 (2014).
