# Supplementary material for: RNA transcripts serve as a template for double-strand break repair in human cells
Source: Nat Commun. 2025 May 10;16:4349. doi: 10.1038/s41467-025-59510-x (PMC12065846; doi:10.1038/s41467-025-59510-x)
Supplement: Supplementary file 2 — Description of Additional Supplementary Files [file 41467_2025_59510_MOESM2_ESM.pdf]

DESCRIPTION OF ADDITIONAL SUPPLEMENTARY FILES

Supplementary data 1

List of all primers and oligos used in this study.
Table 1.1-Probes and primers used for ddPCR of AAVS1-seq.
Table 1.2-Donor oligos used as templates in the assays.
Table 1.3-List of siRNAs used in this study.
Table 1.4-qPCR primers used to quantify gene-expression levels.
Table 1.5-Sequences of the sgRNAs used to in this.
Table 1.6-Primers used for quantification/validation of products.
Table 1.7-Primers used to detect whole intron deletions.

Supplementary data 2

MAGeCK output for CRISPR/Cas9 screen
Table 2.1- MAGeCK output of the CRISPR/Cas9 screen by comparing t=0 versus t=14 to identify essential genes.
Table 2.2- MAGeCK output of screen to identify genes promoting (neg rank) and suppressing (pos rank) SSTR via the DNA^{GFP} donor.
Table 2.3- MAGeCK output of screen to identify promoting (neg rank) and suppressing (pos rank) RT-DSBR via the DNA/RNA^{6R} donor.
All statistical tests were performed by the MAGeCK algorithm, which is cited in the text.

Supplementary data 3

List of all Whole Intron Deletions identified in this study.
Table 3.1-List of WIDs identified in the MSK-IMPACT database.
Table 3.2-Gene expression analysis of some of the patients carrying WIDs from the MSK-IMPACT database.
Table 3.3-List of WIDs identified in the PCAWG database.
