# Supplementary material for: RNA transcripts serve as a template for double-strand break repair in human cells
Source: Nat Commun. 2025 May 10;16:4349. doi: 10.1038/s41467-025-59510-x (PMC12065846; doi:10.1038/s41467-025-59510-x)
Supplement: Supplementary file 6 — Reporting Summary [file 41467_2025_59510_MOESM6_ESM.pdf]

nature portfolio

Corresponding author(s): Sfeir, Agnel and Powell, Simon

Last updated by author(s): Mar 25, 2025

Reporting Summary

Nature Portfolio wishes to improve the reproducibility of the work that we publish. This form provides structure for consistency and transparency in reporting. For further information on Nature Portfolio policies, see our Editorial Policies and the Editorial Policy Checklist.

Statistics

For all statistical analyses, confirm that the following items are present in the figure legend, table legend, main text, or Methods section.

n/a	Confirmed	
☐	☒	The exact sample size (*n*) for each experimental group/condition, given as a discrete number and unit of measurement
☐	☒	A statement on whether measurements were taken from distinct samples or whether the same sample was measured repeatedly
☐	☒	The statistical test(s) used AND whether they are one- or two-sided<br>*Only common tests should be described solely by name; describe more complex techniques in the Methods section.*
☒	☐	A description of all covariates tested
☐	☒	A description of any assumptions or corrections, such as tests of normality and adjustment for multiple comparisons
☐	☒	A full description of the statistical parameters including central tendency (e.g. means) or other basic estimates (e.g. regression coefficient) AND variation (e.g. standard deviation) or associated estimates of uncertainty (e.g. confidence intervals)
☐	☒	For null hypothesis testing, the test statistic (e.g. *F*, *t*, *r*) with confidence intervals, effect sizes, degrees of freedom and *P* value noted<br>*Give P values as exact values whenever suitable.*
☒	☐	For Bayesian analysis, information on the choice of priors and Markov chain Monte Carlo settings
☐	☒	For hierarchical and complex designs, identification of the appropriate level for tests and full reporting of outcomes
☐	☒	Estimates of effect sizes (e.g. Cohen's *d*, Pearson's *r*), indicating how they were calculated

Our web collection on statistics for biologists contains articles on many of the points above.

Software and code

Policy information about availability of computer code

Data collection	BD LSRFortessa™ Cell Analyzer (BD Biosciences)<br>BD FACSDiva™ Software (BD Biosciences) and analyzed using FlowJo v.10 (TreeStar)<br>GE Typhoon FLA 9000 Gel Scanner<br>Bio-Rad ChemiDoc MP Imaging System<br>Bio-Rad ImageLab v.5.2<br>NovaSeq 6000/NovaSeq X<br>GraphPad Prism 9.4.0 http://www.graphpad.com
Data analysis	CRISPResso2 (https://github.com/pinellolab/CRISPResso2.) for AAVS1-seq and Intron-loss assay analysis.<br>Flow cytometry data were analyzed using BD FlowJo (v.10.6.2).<br>GraphPad Prism v9.4.0 was used for data plotting and statistical calculations.<br>All script used to analyze Whole Intron Deletions (WIDs) can be obtained from https://github.com/Yingjie848/project-RNA-templated-DSB-repair-public.<br>RNA sequencing reads were first examined using FASTQC(Andrews 2010), then Illumina universal adapters were trimmed by cutadapt(Martin 2011). The trimmed reads were aligned to the GRCh37 human genome using STAR RNA-Seq aligner(Dobin et al. 2013), and then mapped single-end reads from transcripts were counted using GenomicAlignments package in Bioconductor(Lawrence et al. 2013; Gentleman et al. 2004). Read counts were further transformed into transcripts per million (TPM) normalized for gene length.

For manuscripts utilizing custom algorithms or software that are central to the research but not yet described in published literature, software must be made available to editors and reviewers. We strongly encourage code deposition in a community repository (e.g. GitHub). See the Nature Portfolio guidelines for submitting code & software for further information.

Data

Policy information about availability of data

All manuscripts must include a data availability statement. This statement should provide the following information, where applicable:

- Accession codes, unique identifiers, or web links for publicly available datasets
- A description of any restrictions on data availability
- For clinical datasets or third party data, please ensure that the statement adheres to our policy

The data collected and used in this study are available under:
-FASTQ files from the AAVS1-seq assay: BioProject PRJNA1236828
-RNA-seq of WID tumors: GEO accession GSE291098
-FASTQ files from the Intron-loss assay: GEO accession GSE290535
-Cancer mutation data: Cancer mutation data were retrieved from the MSK-IMPACT and PCAWG databases.
-All scripts used in processing Whole Intron Deletions can be obtained at https://github.com/Yingjie848/project-RNA-templated-DSB-repair-public.

Research involving human participants, their data, or biological material

Policy information about studies with human participants or human data. See also policy information about sex, gender (identity/presentation), and sexual orientation and race, ethnicity and racism.

Reporting on sex and gender	Sex and gender were not considered in the study design
Reporting on race, ethnicity, or other socially relevant groupings	No race, ethnicity of other socially relevant groups were used in this study
Population characteristics	Cancer samples found to harbor WID-containing genes were subjected to PCR amplification and sequence analysis to confirm the presence of WIDs as well as RNA-seq analysis to determine whether the WID containing genes were actively transcribed.
Recruitment	As per conditions of the IRB-protocol
Ethics oversight	The IRB protocol was approved by Memorial Sloan Kettering Cancer Center

Note that full information on the approval of the study protocol must also be provided in the manuscript.

Field-specific reporting

Please select the one below that is the best fit for your research. If you are not sure, read the appropriate sections before making your selection.

☒ Life sciences ☐ Behavioural & social sciences ☐ Ecological, evolutionary & environmental sciences

For a reference copy of the document with all sections, see nature.com/documents/nr-reporting-summary-flat.pdf

Life sciences study design

All studies must disclose on these points even when the disclosure is negative.

Sample size	Sample sizes were always chosen to be large enough for each condition and biological replicate in our experiments to minimize stochastic effects and ensure reproducibility of the data. Exact sample size mentioned for each experiment in the figure legend.
Data exclusions	No data exclusion was applied
Replication	Biological replicates were used
Randomization	Cell samples were always chosen randomly to avoid possible bias
Blinding	No blinding was performed as blinding was not relevant for the study.

Reporting for specific materials, systems and methods

We require information from authors about some types of materials, experimental systems and methods used in many studies. Here, indicate whether each material, system or method listed is relevant to your study. If you are not sure if a list item applies to your research, read the appropriate section before selecting a response.

Materials & experimental systems

n/a	Involved in the study	
☐	☒	Antibodies
☐	☒	Eukaryotic cell lines
☒	☐	Palaeontology and archaeology
☒	☐	Animals and other organisms
☐	☒	Clinical data
☒	☐	Dual use research of concern
☒	☐	Plants

Methods

n/a	Involved in the study	
☒	☐	ChIP-seq
☐	☒	Flow cytometry
☒	☐	MRI-based neuroimaging

Antibodies

Antibodies used

FLAG (Clone M2, Sigma; 1: 10000 dilution)
53BP1 (NB100-304, Novus Biologicals; 1:1000 dilution)
HNRNPK (sc-28380, Santa Cruz; 1:1000 dilution)
HNRNPC (sc-32308, Santa Cruz; 1:1000 dilution)
GAPDH (0411, Santa Cruz, 1:10000 dilution)
vinculin (13901, Cell Signaling; 1:1000 dilution)
γ-tubulin (GTU-88; Sigma Aldrich; 1:5000 dilution)
mouse IgG HRP-linked (NA931, GE Healthcare; 1:5000)
rabbit IgG HRP-linked (NA934, GE Healthcare; 1:5000)

Validation

Antibodies were validated by the manufacturer.

Eukaryotic cell lines

Policy information about cell lines and Sex and Gender in Research

Cell line source(s)

HEK293T (ATCC, #CLR-3216)

Authentication

STR profiling is used to authenticate the Hek293T cell lines.

Mycoplasma contamination

Cell lines were regularly tested for mycoplasma contamination

Commonly misidentified lines
(See ICLAC register)

No commonly misidentified lines were used.

Clinical data

Policy information about clinical studies
All manuscripts should comply with the ICMJE guidelines for publication of clinical research and a completed CONSORT checklist must be included with all submissions.

Clinical trial registration

n/a

Study protocol

12-245; PCAWG

Data collection

tumor DNA for MSK IMPACT sequencing; tumor DNA for whole genome sequencing; tumor RNA for RNA-seq

Outcomes

n/a

Plants

Seed stocks

n/a

Novel plant genotypes

n/a

Authentication

n/a

Flow Cytometry

Plots

Confirm that:

☒ The axis labels state the marker and fluorochrome used (e.g. CD4-FITC).

☒ The axis scales are clearly visible. Include numbers along axes only for bottom left plot of group (a 'group' is an analysis of identical markers).

☒ All plots are contour plots with outliers or pseudocolor plots.

☒ A numerical value for number of cells or percentage (with statistics) is provided.

Methodology

Sample preparation	Two million cells were collected by trypsinization, washed with cold PBS, and resuspended in PBS with 3% of FBS. Data on fluorescent content were acquired on a BD LSRFortessa™ Cell Analyzer (BD Biosciences) using BD FACSDiva™ Software (BD Biosciences) and analyzed using FlowJo v.10 (TreeStar).
Instrument	BD LSRFortessa™ Cell Analyzer (BD Biosciences)
Software	BD FACSDiva™ Software (BD Biosciences) and analyzed using FlowJo v.10 (TreeStar).
Cell population abundance	A viability die (propidium iodide) was used to exclude dead cells.
Gating strategy	Dead cells and doublet were excluded from the analysis.

☒ Tick this box to confirm that a figure exemplifying the gating strategy is provided in the Supplementary Information.
